# P-1134. Hospital Sink Drains and Wastewater Harbor Distinct Yet Overlapping Multidrug-Resistant Bacterial Species and Resistance Gene Plasmids

**DOI:** 10.1093/ofid/ofaf695.1328

**Published:** 2026-01-11

**Authors:** Medini Annavajhala, Kasiani Terzoglou, Todd Hokunson, Anne-Catrin Uhlemann, Angela Gomez-Simmonds

**Affiliations:** Children's Hospital of Philadelphia, Philadelphia, Pennsylvania; Children's Hospital of Philadelphia, Philadelphia, Pennsylvania; Columbia University Irving Medical Center, New York, New York; Columbia University Medical Center, New York, New York; University of California, Davis, Davis, CA

## Abstract

**Background:**

Multidrug-resistant bacteria (MDRB) have been shown to disseminate and persist within the hospital environment, potentially increasing risk of infection in hospitalized patients. Extended spectrum beta-lactamase-producing and carbapenem-resistant Enterobacterales (ESBLE, CRE) have been identified in hospital sinks and wastewater (WW) primarily as part of outbreak investigations, but longitudinal studies are lacking.

**Figure 1.**
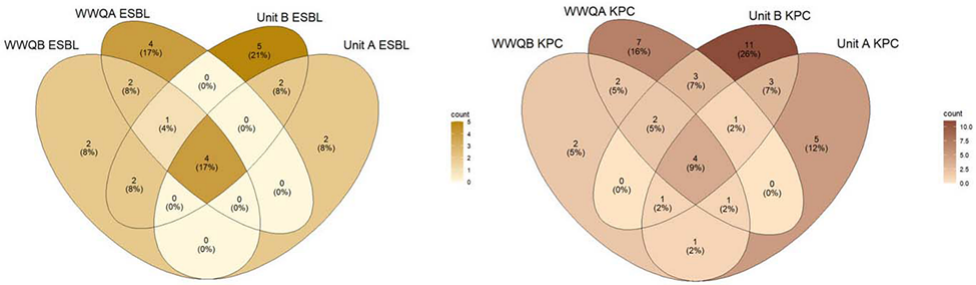
Venn diagrams of (left) ESBLE and (right) CRE species identified in sink drains from two hospital units (Unit A and Unit B) and hospital wastewater collected from each unit (WWQA and WWQB) demonstrated the presence of unique ESBLE and CRE species across sample types.

**Figure 2.**
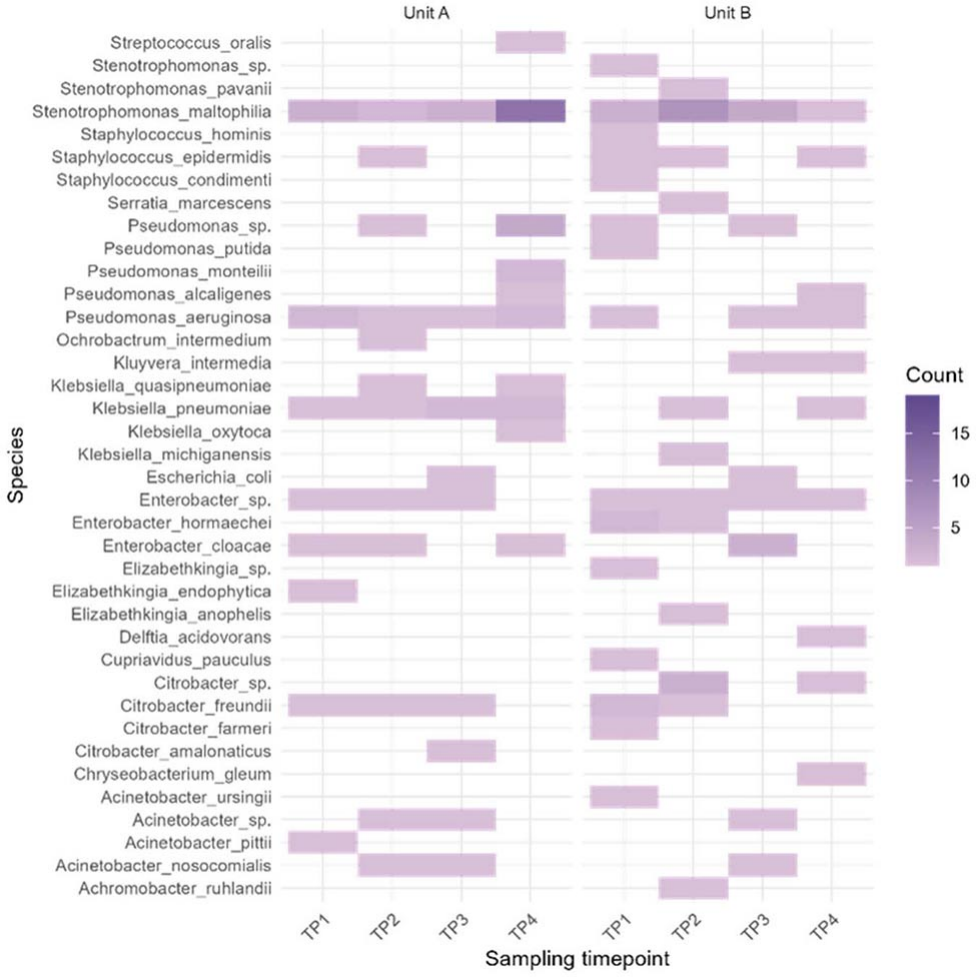
Heatmap showing the distribution, sampling frequency, and persistence of bacterial species across sampling timepoints for Units A and B. Here counts refer to the number of isolates recovered per sampling timepoint for a given bacterial species in each unit.

**Methods:**

We performed environmental surveillance for ESBLE and CRE at a New York City hospital over a six-month period in 2023. Swabs were collected from sink drains in two inpatient hospital units (Units A, B) over four sampling timepoints and concurrent sampling was implemented from WW traps linked to each of these patient units (Quadrants A [WWQA], B [WWQB]). ESBLE and CRE colonies were isolated using ESBL and KPC CHROMagar plates, respectively, and underwent nanopore whole genome sequencing. Assembled genomes were used for comparative genomic analyses to identify links between sampling sites, across timepoints, and with clinical MDRB.

**Results:**

Overall, we isolated 52 ESBLE and 211 CRE from 317 sink swabs and 140 ESBLE and 168 CRE from 135 WW samples. Overall, distinct bacterial species were identified in sink drain and WW samples, between Units A and B, and between WWQA and WWQB (Fig 1), suggesting differential intra-hospital environmental colonization. In sink drains, several species including *Stenotrophomonas maltophilia* were identifed in both hospital units and persisted across timepoints (Fig 2), while others such as *Klebsiella pneumoniae* differed in prevalence across units. *E. coli* (n=51, 31%) and *Raoultella ornithinolytica* (n=45, 19%) were the most commonly identified ESBLE and CRE in WW samples, respectively, despite low prevalence in drains. Close phylogenetic links (< 25 SNPs) were observed between environmental and clinical isolates (n=435), particularly within *Enterobacter cloacae* complex and *K. pneumoniae*. Clustering analysis also revealed resistance plasmids shared between sink drain, WW, and clinical isolates and unrelated MDRB.

**Conclusion:**

In summary, in a culture-based analysis, CRE and ESBLE demonstrated longitudinal colonization of the hospital environment but MDRB species composition differed between sink drain and WW sites.

**Disclosures:**

Anne-Catrin Uhlemann, MD, PhD, Merck: Grant/Research Support

